# MDA-9/Syntenin (SDCBP) Is a Critical Regulator of Chemoresistance, Survival and Stemness in Prostate Cancer Stem Cells

**DOI:** 10.3390/cancers12010053

**Published:** 2019-12-23

**Authors:** Sarmistha Talukdar, Swadesh K. Das, Anjan K. Pradhan, Luni Emdad, Jolene J. Windle, Devanand Sarkar, Paul B. Fisher

**Affiliations:** 1 Department of Human and Molecular Genetics, School of Medicine, Virginia Commonwealth University, Richmond, VA 23298, USA; sarmistha.talukdar@gmail.com (S.T.); swadesh.das@vcuhealth.org (S.K.D.); anjan.pradhan@vcuhealth.org (A.K.P.); luni.emdad@vcuhealth.org (L.E.); jolene.windle@vcuhealth.org (J.J.W.); devanand.sarkar@vcuhealth.org (D.S.); 2 VCU Institute of Molecular Medicine, School of Medicine, Virginia Commonwealth University, Richmond, VA 23298, USA; 3 VCU Massey Cancer Center, School of Medicine, Virginia Commonwealth University, Richmond, VA 23298, USA

**Keywords:** MDA-9/Syntenin (SDCBP), stemness, prostate cancer stem cells, survival, apoptosis, chemoresistance

## Abstract

Despite some progress, treating advanced prostate cancer remains a major clinical challenge. Recent studies have shown that prostate cancer can originate from undifferentiated, rare, stem cell-like populations within the heterogeneous tumor mass, which play seminal roles in tumor formation, maintenance of tumor homeostasis and initiation of metastases. These cells possess enhanced propensity toward chemoresistance and may serve as a prognostic factor for prostate cancer recurrence. Despite extensive studies, selective targeted therapies against these stem cell-like populations are limited and more detailed experiments are required to develop novel targeted therapeutics. We now show that MDA-9/Syntenin/SDCBP (MDA-9) is a critical regulator of survival, stemness and chemoresistance in prostate cancer stem cells (PCSCs). MDA-9 regulates the expression of multiple stem-regulatory genes and loss of MDA-9 causes a complete collapse of the stem-regulatory network in PCSCs. Loss of MDA-9 also sensitizes PCSCs to multiple chemotherapeutics with different modes of action, such as docetaxel and trichostatin-A, suggesting that MDA-9 may regulate multiple drug resistance. Mechanistically, MDA-9-mediated multiple drug resistance, stemness and survival are regulated in PCSCs through activation of STAT3. Activated STAT3 regulates chemoresistance in PCSCs through protective autophagy as well as regulation of MDR1 on the surface of the PCSCs. We now demonstrate that MDA-9 is a critical regulator of PCSC survival and stemness via exploiting the inter-connected STAT3 and *c-myc* pathways.

## 1. Introduction 

Prostate cancer is the most common male cancer and the second principal cause of cancer-associated deaths among men [1]. Despite recent progress in detecting prostate cancer early, efficacious therapies for late stage disease remain ineffective and limited. The main treatment strategy for localized prostate cancer is either radiotherapy (external beam or brachytherapy) or radical prostatectomy. Additionally, androgen deprivation and androgen receptor-targeted therapies [2] are frequently used in combination with surgery and/or radiotherapy. In spite of these therapeutic strategies, 30%–50% of intermediate- to high-risk patients relapse and neoplastic growth of aberrant prostate cells resumes either locally or systematically [3]. Moreover, the therapeutic strategies for patients with advanced and/or metastatic disease have little to no curative effect [4].

Prostate cancer stem cells (PCSCs) play a seminal role in the initiation, progression, and resistance to therapy of prostate cancer and consequently represent a principal cause of treatment failure [5,6,7,8]. Several reports document the existence of this unique stem cell subpopulation in prostate cancer [8,9]. Prostate homeostasis is maintained as a result of physiological factors, including external stimuli, and androgenic regulation of stromal-epithelial interactions, where an imbalance in this niche can contribute to malignancy [4]. Prostate epithelial stem cells are located on the basement membrane of the adult prostate, positioned in their niche due to high integrin α2β1 expression [4,10]. A basal/luminal cell origin for prostate cancer has been suggested [11]. Despite similarities, there are some differences between normal and cancer prostate stem cells. Unlike normal prostate stem cells, PCSCs are slow-cycling cells and do not have a static population [11]. Cell surface molecules such as β1integrins, NOTCH1, DLL1, and IGF-R1 provide important signals for many niches, triggering several downstream signaling pathways essential for prostate cancer progression, stemness and survival [4,12,13,14,15,16].

The NOTCH signaling pathway is involved in vascular development as well as cancer stem cell maintenance, emphasizing the significance of CSC-mediated angiogenesis [17]. Gene expression profiling emphasizes the importance of JAK-STAT, C-myc, and Wnt over-expression in PCSCs [4,18,19]. Among the isolated populations from primary prostate cancers, only CD44^+^ CD133^+^ cells display in vitro self-renewal [4,9,10] and express core stem cell genes OCT4, NANOG, SOX2, nestin, and c-kit [4,20]. CD44, a glycoprotein involved in cell–cell interactions, cell adhesion and migration, is a marker of stemness of CSCs for several cancer types, including prostate [21,22]. Unlike CD44^−^ cells, CD44^+^ prostate cancer cells from xenografted human tumors are enriched in tumorigenic and metastatic progenitor cells [22,23]. CD133 is also accepted as a CSC cell surface marker for several cancers, including prostate cancer [22,24]. CD133^+^ cells have elevated in vitro proliferative potential with the ability to form prostatic-like acini in immunocompromised male nude mice [22,25].

Signal transducer and activator of transcription (STAT) proteins, predominantly STAT3 proteins, are implicated in prostate tumorigenesis [11,26,27]. Activated STAT3 correlates with a higher Gleason score, pathological stage of prostate cancer, decreased survival [11,28,29,30], and shorter time to death as a consequence of biochemical relapse [11,29]. Recurrence-free survival rates are also lower in patients displaying increased levels of STAT3 activation [11,29]. While STAT3 activation has been extensively studied in prostate cancer development, its role in PCSCs has only recently been investigated. Pre-treating human PCSCs with increasing concentrations of a STAT3 inhibitor abrogated in vivo tumor-propagating ability [11,31]. Human prostaspheres display elevated levels of STAT3 activation [11,32,33] and STAT3 knockdown leads to reduced sphere formation and in vivo tumor growth [11,34]. PCSCs with their enhanced development of radioresistance, chemoresistance [35,36], and castration-resistant disease provide adaptive or selective pressure for increasing their number and/or activity [11]. The main mechanism by which cancer stem cells protect themselves is through the expression of ATP-binding cassette or ABC transporters (ABCB1/P-glycoprotein/MDR1, ABCC1, ABCG2, etc.). These transporters pump out cytotoxic drugs and function as the guardians of the stem cell population [37]. Human P-glycoprotein is encoded by the multidrug resistance 1 (MDR1) gene, located on chromosome 7q21, that functions in the transport and/or efflux of its substrates, thereby defending tissues from physiologically damaging substances, cytotoxic agents, and xenobiotics [37]. Unfortunately, these ABC efflux pumps also provide protection of cancer stem cells in tumors from the cytotoxic effects of chemotherapy [38]. Additionally, MDR1 expression is significantly elevated in drug-resistant tumors [39,40]. Such signatures of CSCs as well as their number may be a prognostic factor for prostate cancer recurrence [41]. PCSCs are also associated with medium Gleason grades, when the tumor is still confined to the prostate gland. At this stage, surgery is usually performed with curative intent; however, a significant percentage of patients relapse after treatment [11]. Chemotherapeutic treatments often lead to the selection of resistant cancer cells and CSCs are known to closely cooperate with endothelial precursor cells, leading to the formation of a vascular/cancer stem niche, through overlapping mechanisms and responses [17].

In spite of recent surge of studies, it is clear that PCSCs need more detailed studies which bring new molecular targets to attention and that without an understanding of the sequence of complex molecular interactions, the stochastic and hierarchical cancer stem cell models, targeting these cells is not possible [4].

MDA-9 is a widely distributed cytosolic protein that interacts with a gamut of crucial regulatory proteins, such as SRC, FAK and EGFR, through its amino, carboxy- and PDZ domains, thereby contributing significantly to cancer evolution [42,43,44,45,46]. MDA-9 plays a pivotal role in cancer progression and recent studies indicated that it can serve as a diagnostic marker of tumor aggression and grade in several cancer types [42,43,44,47,48]. Based on these observations, we hypothesized that higher tumor grade, which correlates with a more invasive and metastatic phenotype, might contain an increased proportion of PCSCs expressing elevated levels of MDA-9. We presently assessed the association between stemness and MDA-9 expression in prostate cancer, as well as in normal prostate epithelial cells. Self-renewal ability of putative PCSCs was evaluated utilizing sphere forming assays, cell-surface stem marker expression, and detailed studies of molecules regulating self-renewal, stem cell maintenance and tumorigenicity. Additionally, the effects of MDA-9 on PCSC survival, proliferation and chemoresistance were examined. The mechanisms responsible for MDA-9-mediated PCSC phenotype, maintenance, viability and chemoresistance were also scrutinized. Our experiments establish MDA-9 as a critical regulator of stem cell phenotypes in prostate cancer that are responsible for PCSC maintenance and survival through regulation of multiple stem-regulating molecules such as NOTCH1, C-myc, STAT3, NANOG, OCT4 and SOX2. Additionally, MDA-9 regulates resistance of PCSCs to multiple chemotherapeutic drugs used in prostate cancer treatment. These multifaceted roles of MDA-9 in prostate cancer provide an opportunity to use CSC-based theranostic approaches that target this gene and its protein for effective diagnosis and therapy of PCSCs.

## 2. Results

### 2.1. MDA-9 Expression Is Elevated in the Unique Self-Renewing PCSC Subpopulation in Prostate Cancer

PCSCs from different prostate cancer cell lines (DU-145, ARCaP-M and PC3-ML) were sorted into putative CD44^+^ CD133^+^ PCSCs and CD44^−^ CD133^−^ non-stem cancer cells (NSCCs). *mda-9* expression was analyzed in these putative stem and non-stem cancer cells by quantitative RT-PCR, and data were normalized to 18S and β-tubulin expression. We consistently observed elevated expression of *mda-9* in all PCSC populations vs. NSCCs (Table 1). These PCSCs also expressed high levels of traditional stem-regulatory and self-renewal associated genes such as *Nanog*, *Oct4*, *Sox2* and *c-myc* (Table 1).

### 2.2. mda-9 Is Co-Expressed in PCSCs with Stem Cell Markers and Stemness-Regulating Genes 

**Figure 1:**
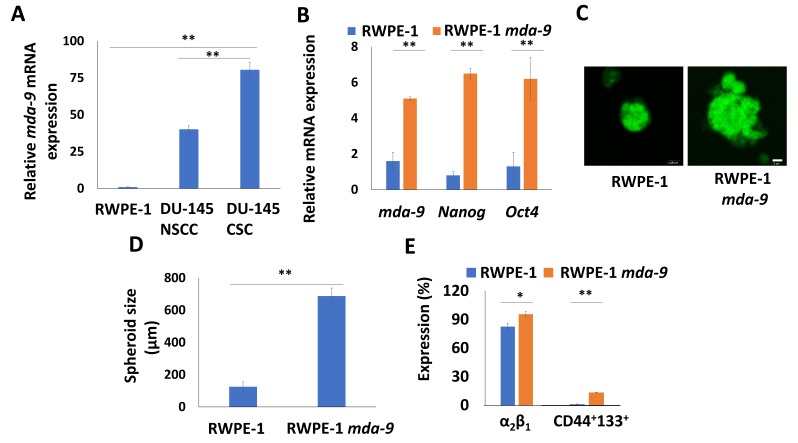
*mda-9* Expression correlates with stemness markers. (**A**) Expression of *mda-9* in normal prostate and prostate cancer stem cells. (**B**) Expression of *Nanog,* and *Oct4* in *mda-9* overexpressing normal prostate stem cells. (**C**) Confocal image showing the size of RWPE-1 cells in parental and *mda-9* overexpressing prostaspheres. (**D**) Graphical depiction of the spheroid size in RWPE-1 prostaspheres in parental and *mda-9* overexpressing cells. (**E**) Effect of *mda-9* overexpression on normal prostate stem cell populations. The scale bars represent 20 µm. * *p* < 0.05, ** *p* < 0.01, using the Student’s *t*-test and ANOVA.

Normalized relative gene expression from PCSCs isolated from different cell lines was analyzed statistically by Pearson’s correlation coefficient and ANOVA analysis for correlation and significance, respectively. A positive correlation was observed between *mda-9* expression and stemness genes, including *mda-9*: *Nanog*, (Pearson’s correlation coefficient *R* = 0.7303), *mda-9:Sox2* (*R* = 0.6881), *mda-9:Oct4* (*R* = 0.4241), *mda-9:c-myc* (*R* = 0.7279). The results were statistically significant (*R*^2^ = 0.7825, *p* < 0.05) and the strongest correlation was observed between *mda-9*, *Nanog* and *c-myc*. *mda-9* expression in DU-145 cancer cells was also several-fold higher than in normal prostate stem cells, with the highest expression being observed in cancer stem cells (Figure 1A).

### 2.3. MDA-9 Over-Expression Leads to Expression of a Stem-Like Phenotype 

Forced expression of *mda-9* in normal prostate non-stem cells lead to increased expression of self-renewal genes such as *Nanog* and *Oct4* (~6 fold) compared to that of parental cells (Figure 1B). When the stem populations (stained with green fluorescent cell tracker) in the prostaspheres were studied, a significant increase in spheroid size, and number was observed (Figure 1C,D). *mda-9* overexpression also increased stem populations, as demonstrated by a cell-surface marker-based flowcytometry analysis (Figure 1E and Supplementary Figure S1A). Overexpression of *mda-9* in the non-stem cancer cells of DU-145 and PC3-ML also led to an approximately 2–4-fold increase in PCSCs as well as self-renewal associated genes (*Nanog* ~13–22-fold, *Sox2* ~2–6-fold, *Oct4* ~6.8–15-fold) (Supplementary Figure S1B). These results indicate that MDA-9 may have a central role in the regulation of self-renewal in both normal and malignant prostate cells.

### 2.4. MDA-9 Activates Downstream Signaling, Which Regulates Self-Renewal in PCSCs

To further ascertain the role of MDA-9 in regulating PCSC self-renewal and maintenance, we silenced *mda-9* in PCSCs from DU-145, ARCaP-M and PC3-ML. Knocking down *mda-9* in PCSCs significantly decreased the population of PCSCs (Table 2) as well as expression of self-renewal associated molecules at both RNA (Table 3) and protein levels (Table 4). *Nanog* was decreased by almost two-fold, ~10-fold and ~6.7-fold, *Sox2* by ~three-fold, ~four-fold and ~10-fold, *Oct4* by ~seven-fold, ~10-fold and ~33-fold, and *c-myc* by ~six-fold, ~20-fold and ~11-fold, in DU-145, ARCaP-M and PC3-ML PCSCs, respectively, post *mda-9* knock down (kd). These results suggest that *mda-9* expression is crucial in maintaining expression of self-renewal associated genes in PCSCs.

Considering the significant role of STAT3 in prostate cancer [49] and PCSCs [34], we analyzed the effect of MDA-9 on STAT3 in PCSCs. *mda-9* kd significantly decreased p-STAT3 (Tyr-705) expression by ~1.5–3.5-fold in DU-145, ARCaP-M and PC3-ML PCSCs (Table 4). Active SRC is also known to positively regulate STAT3 [45] and we found significant decreases ranging from ~1.5–6-fold, in SRC activation post *mda-9* kd (Table 4). STAT3 is additionally regulated by p44/42 and IGF-1R [50,51,52], and considering this, we also examined the expression of these proteins in control and sh*mda-9* PCSCs. A significant decrease in p44/42 was evident (Table 4), and an even more profound decrease in phospho-p44/42 (Table 4). p-IGF-1R was also significantly decreased in the sh*mda-9* cells (Table 4). STAT3 is required for *c-myc* expression [53,54,55], which potentially adds another key regulatory element involved in PCSC maintenance.

### 2.5. MDA-9 Maintains PCSC-Mediated Survival and Tumorigenicity 

**Figure 2:**
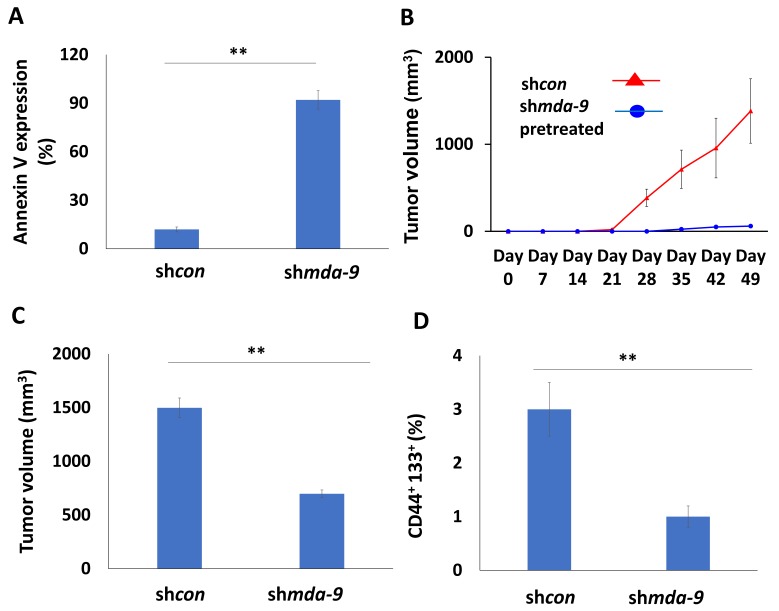
MDA-9 regulates survival and tumorigenic potential in PCSCs. (**A**) Effect of *mda-9* kd in promoting PCSCs cell death analyzed by Annexin V assays. (**B**) Effect of *mda-9* kd on PCSC tumorigenicity (*n* = 10). (**C**) Effect of intra-tumoral *mda-9* kd on tumorigenicity (*n* = 10). (**D**) PCSC populations in tumors following intra-tumoral *mda-9* kd. The bars represent SEM. ** *p* < 0.01 using the Student’s *t*-test and ANOVA.

**Figure 3:**
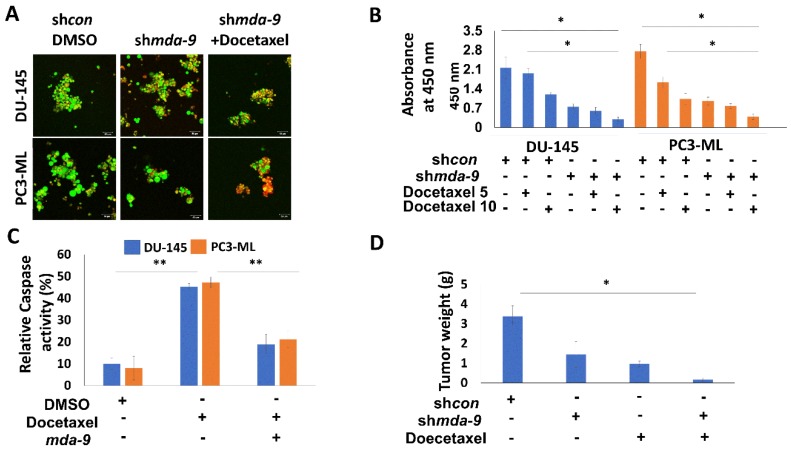
*mda-9* regulates chemoresistance in PCSCs. (**A**) Effect of *mda-9* kd on PCSC sensitivity to Docetaxel assessed by Live/Dead assay: live cells are green, dead cells are red. (**B**) Effect of *mda-9* kd on PCSC sensitivity to Docetaxel (5 and 10 nM), assessed by MTT assay. (**C**) Effect of *mda-9* overexpression on PCSC sensitivity to Docetaxel assessed by caspase activity. (**D**) Effect of intra-tumoral *mda-9* kd on Docetaxel sensitivity measured by tumor weight (*n* = 10). The bars represent SEM. * *p* < 0.05, ** *p* < 0.01, using the Student’s *t*-test and ANOVA.

Apart from the loss of self-renewal (Table 2), *mda-9* kd also significantly increases cell death and apoptosis in PCSCs from DU-145 cells, as early as 72 h post kd (Figure 2A and Figure S2A). *mda-9* kd in PCSCs decreased tumorigenicity. The pretreated sh*mda-9* cells were obtained by treating PCSCs with Ad.5/3.sh*mda-9* at 1000 v.p. per cell. When sh*con* and sh*mda-9* pre-treated DU-145 PCSCs were injected subcutaneously into male nude mice (*n* = 10), the sh*con* group formed large tumors with a substantial population of PCSCs (Figure 2B). However, the sh*mda-9* tumors were extremely small (Figure 2B). *mda-9* silencing with intra-tumoral injection of sh*mda-9* virus in subcutaneous DU-145 PCSC-derived tumors also resulted in smaller tumors, with decreased tumor growth kinetics and PCSC populations in the intra-tumoral treated groups (Figure 2C,D). The PCSCs are grown in anoikis conditions *in vitro*, and these cells are sensitive to anoikis post *mda-9* kd [46]. This may be the reason why pretreated PCSCs did not form tumors, but intra-tumoral *mda-9* kd PCSCs were able to form small tumors. However, the results show that MDA-9 is vital for PCSC function, and the loss of MDA-9 leads to a sharp decrease in their ability to form tumors. MDA-9 is also essential for PCSC survival and maintenance of the CSC-niche, as loss of MDA-9 leads to loss of spheroid integrity, and ultimately, loss of PCSC viability (Figure 3A). 

### 2.6. Suppression of MDA-9 Sensitizes PCSCs to Multiple Chemotherapeutic Drugs via STAT3 

**Figure 4:**
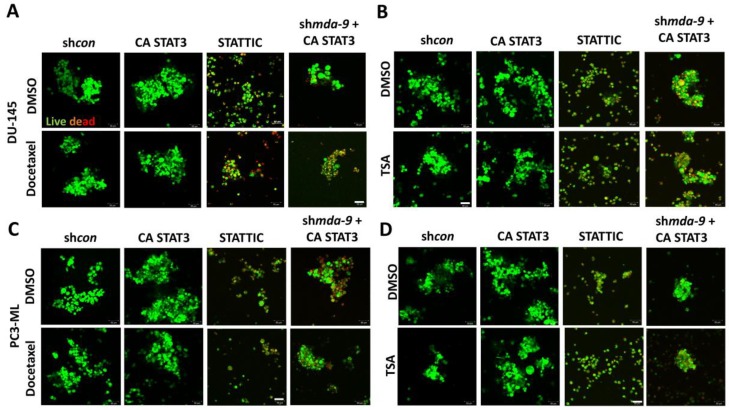
STAT3 activation is downstream of *mda-9* and it regulates PCSC chemoresistance. Image analysis of sh*con* or *mda-9* kd (sh*mda-9*) PCSCs overexpressing constitutively active (CA) STAT3 or treated with STAT3 inhibitor STATTIC; (**A**) of DU-145 treated with or without Docetaxel, (**B**) of DU-145 treated with or without TSA (**C**) of PC3-ML treated with or without Docetaxel (**D**) of PC3-ML treated with or without TSA, live cells are green, dead cells are red.

PCSCs are relatively resistant to docetaxel (Figure 3A,B) resulting in a range of ~65%–70% cell proliferation vs. control post 10 and 5 nM treatment (Figure 3B). However, the sh*mda-9* PCSCs show a range of ~35%–40% and ~20%–30% cell proliferation vs. control following 10 and 5 nM treatment, respectively (Figure 3B). Overexpression of *mda-9* led to a decrease in docetaxel and trichostatin-A-induced caspase activity (Figure 3C and Supplementary Figure S2B). These results indicate that the kd of MDA-9 results in sensitivity of PCSCs to docetaxel and trichostatin-A, and that the high expression of MDA-9 in the PCSCs may confer some chemoresistance to these unique populations of cells. *In vivo* xenograft studies also confirmed that loss of MDA-9 by both genetic and pharmacological techniques lead to sensitization to docetaxel treatment (Figure 3D and Supplementary Figure S2C,D). Further experiments suggested that MDA-9 regulates this resistance phenotype through STAT3 activation. This was confirmed through CA-STAT3 (constitutively active STAT3) overexpression and STAT3 inhibitor studies (STATTIC). We overexpressed a constitutively active STAT3 (A662C/N664C; *CA-STAT3*) in the sh*mda-9* PCSCs and observed a recovery of resistance to Docetaxel and Trichostatin A (Figure 4 and Supplementary Figure S3A). Interestingly, when we used a non-constitutively active STAT3, this rescue effect was abrogated (Supplementary Figure S3A). CA-STAT3 was able to confer chemoresistance in *shmda-9* PCSCs, whereas STATTIC caused chemosensitivity to both docetaxel and trichostatin-A (Figure 4 and Supplementary Figure S3C). Additionally, overexpression of CA-STAT3 in the sh*mda-9* PCSCs abrogated the inhibitory effects of *mda-9* kd on expression of stemness regulatory genes (Supplementary Figure S3B). These results confirm that MDA-9 exerts a stemness and chemoresistance phenotype in PCSCs by regulating STAT3.

### 2.7. MDA-9 Mediates PCSC Chemoresistance through the STAT3-MDR1 Axis

**Figure 5:**
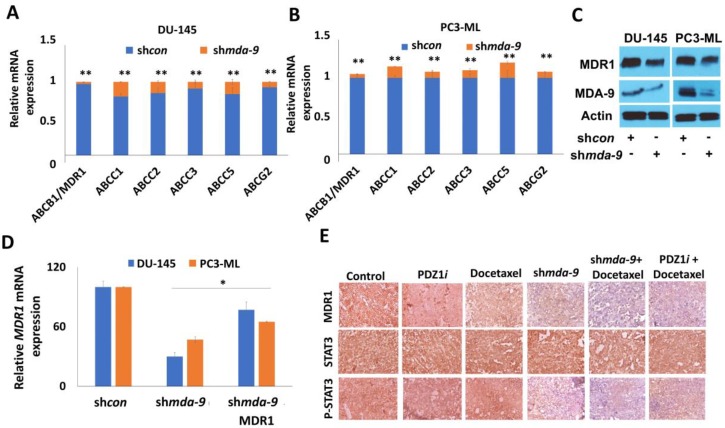
*mda-9* regulates MDR1 expression in PCSCs. RT-PCR analysis to study the effect of *mda-9* kd on ABC family gene expression in (**A**) DU-145 and (**B**) PC3-ML PCSCs. (**C**) Effect of *mda-9* kd on MDR1 protein expression in DU-145 and PC3-ML PCSCs by Western blotting. (**D**) Effect of *mda-9* kd on *MDR1* mRNA expression in DU-145 and PC3-ML PCSCs (**E**) Expression of MDR1, STAT3 and P-STAT3 in control, *mda-9* kd, PDZ1i and Docetaxel treated tumors analyzed by IHC (*n* = 8). The bars represent SEM. * *p* < 0.05, ** *p* < 0.01, using the Student’s *t*-test and ANOVA.

**Figure 6:**
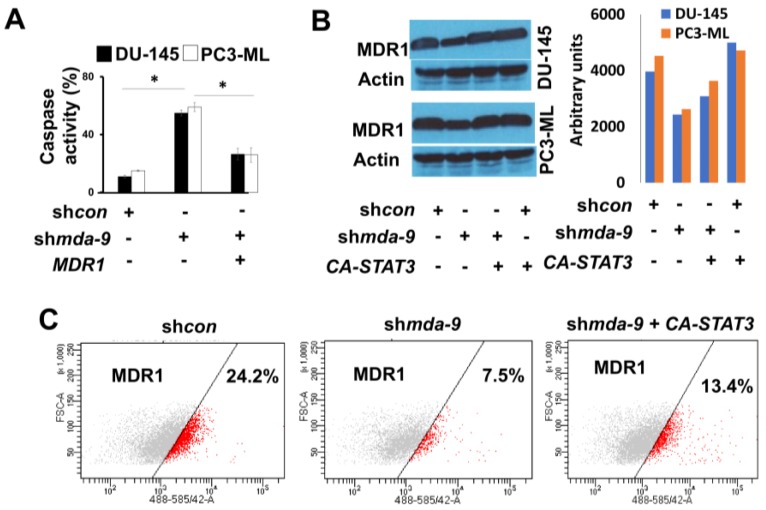
*mda-9* regulates MDR1 expression through STAT3 activation. (**A**) Effect of *MDR1* overexpression on *mda-9* kd mediated caspase activity. (**B**) Effect of *CA-STAT3* overexpression on MDR1 protein expression in DU-145 and PC3-ML PCSCs by Western blotting. (**C**) Effect of *CA-STAT3* overexpression on MDR1 protein expression in ARCaP-M PCSCs by flowcytometry. The bars represent SEM. * *p* < 0.05, using the Student’s *t*-test and ANOVA.

Since we observed a possible role of MDA-9 in PCSC chemoresistance, we analyzed the expression of the ABC family of genes in DU-145 and PC3-ML PCSCs (Figure 5A,B). We observed that the ABC family of genes was highly expressed in both cell lines with ABCB1 or MDR1/P-glycoprotein being the most significantly affected by *mda-9* kd (Figure 5A–C). MDR1 mRNA expression decreased significantly in the sh*mda-9* group as compared to the sh*con* group (~33-fold in DU-145 and ~20-fold in PC3-ML, as shown in Figure 5D). The protein levels of MDR1 also decreased significantly in the sh*mda-9* group as compared to the sh*con* group (DU-145 ~1.7-fold and PC3-ML ~1.6-fold) as shown in Figure 5C and Figure S4A. Protein levels in ARCaP-M sh*mda-9* PCSCs decreased by ~three-fold, as shown in Figure 6C. When we analyzed in vivo mice tumor xenograft histologies, these results were substantiated (Figure 5E). *mda-9* kd in PCSCs resulted in increased apoptotic cell death as evidenced by enhanced caspase 3 activity (Figure 6A), which was rescued by MDR1 overexpression (Figure 5D and Figure 6A and Supplementary Figure S4B). In addition, *mda-9* kd-mediated MDR1 expression loss was significantly rescued by the overexpression of CA-STAT3 in DU-145, PC3-ML and ARCaP-M PCSCs (Figure 6B,C). These results show that MDR1 is important in lessening caspase-mediated cell death in *mda-9* kd-treated cells (Figure 6A). STAT3 plays a very important role in MDA-9-mediated MDR1 expression, thereby contributing to chemoresistance to multiple drugs such as docetaxel and Trichostatin-A (TSA).

### 2.8. C-myc Regulation by MDA-9 Is Essential for Stem Cell Renewal, Maintenance, Survival and MDR1 Expression

The importance of C-myc in prostate cancer has been established [56,57]. Recently, the role of C-myc in stem cell self-renewal, maintenance, and survival [58,59] has being emphasized. To confirm whether MDA-9 contributes to C-myc regulation in PCSCs, we analyzed its expression in sh*con* and sh*mda-9*-treated PCSCs. Suppression of *mda-9* by kd led to a significant decrease in C-myc expression at RNA and protein levels (RNA: ~5.9 to ~47.6-fold change Table 3; protein: ~1.4 to two-fold change as compared to sh*con* (Table 4). Additionally, C-myc inhibition led to a decrease in MDR1 expression as well as chemoresistance to Docetaxel (Figure S5), which was recovered by overexpression of *mda-9*. C-myc expression is regulated by RBPJK via NOTCH1 signaling, and this is possibly the pathway by which MDA-9 mediates C-Myc regulation. NOTCH1 expression consistently decreased following *mda-9* kd in DU-145, ARCaP-M and PC3-ML PCSCs (Table 4). This decrease in NOTCH1 expression in *mda-9* kd PCSCs probably results from elevated NOTCH1 degradation due to the increased expression of NUMB in DU-145, ARCaP-M and PC3-ML PCSCs (Table 4). Utilizing a NOTCH-Blocking Peptide (NBP) to block NOTCH1 activation and downstream signaling also resulted in the same phenotype observed in *mda-9* kd PCSCs (Figure S6). These results indicate that MDA-9 regulates C-myc in PCSCs, through NOTCH1 signaling. Collectively, our findings suggest that MDA-9 regulates MDR1-mediated chemoresistance in PCSCs through C-myc, in addition to STAT3. 

## 3. Discussion

PCSCs play a defining role in prostate cancer initiation and progression by regulating invasion, angiogenesis, metastasis, resistance to therapy and tumor recurrence. PCSCs have significant prognostic value and molecules that are highly expressed in these unique populations can not only shed new light on their functions, but also may serve as potential new targets for prostate cancer therapy [60]. MDA-9 is reported to be diagnostic of both tumor aggression and grade, with a significant positive association between MDA-9 expression and tumor stage in several different cancer types [44,47]. We found that *mda-9* is highly expressed in PCSCs from DU-145, ARCaP-M and PC3-ML, compared to non-stem prostate cancer cells and normal prostate epithelial cells (Table 1, Figure 1A). *mda-9* expression also positively correlated with expression of established self-renewal regulatory genes, including *Nanog, Oct4, Sox2* and *c-myc* (Table 1 and Table 3). Gain and loss of *mda-9* function also correlate with an increase/decrease of PCSC populations and stemness, respectively (Figure 1 and Figure S1; Table 2 and Table 3). Silencing of *mda-9* decreases the activation of STAT3 (Table 4), which is an established regulator of prostate tumorigenesis and metastasis [49,61], as well as the transcription of self-renewal genes [62,63]. These are also the same genes that are upregulated or downregulated following *mda-9* overexpression or silencing, respectively, suggesting that *mda-9* may regulate the traditional self-renewal genes through STAT3. Overexpression of constitutively active STAT3 in the sh*mda-9* PCSCs was able to rescue stem cell phenotype caused by the loss of *mda-9*, confirming our hypothesis. STAT3 is reported to be regulated by SRC, IGF-1R, and p-44/42 [50,51,52]. Phosphorylated p-44/42 (T202/Y204) and SRC can phosphorylate STAT3 (Y705). Our data indicate that MDA-9 may also regulate STAT3 through IGF-1R, p-44/42 and SRC signaling (Table 4). MDA-9 also affects FAK [64,65], RAF and RKIP [66] activity, which are crucial for the activation of p-44/42. In the case of PCSCs, there may be multiple levels of control (Table 3 and Table 4) where MDA-9 can regulate self-renewal via STAT3 activation. 

The NOTCH1 pathway is also essential for PCSC functions [12], and its aberrant signaling is known to promote tumorigenesis [12]. Our data indicate that MDA-9 can also regulate NOTCH1 signaling on multiple levels. DLL1, a ligand of NOTCH1, is essential for the activation of NOTCH1 and its downstream signaling. MDA-9 is crucial for DLL1 recycling in stem cells [45], and suppression of *mda-9* leads to loss of cell-surface expression of DLL1 (Table 4). This results in decreased NOTCH1 signaling. sh*mda-9* PCSCS also have decreased expression of p-SRC, and increased expression of NUMB, which are positive and negative regulators of NOTCH1, respectively (Table 4). This may explain the decreased cell-surface expression of NOTCH1 on the sh*mda-9* PCSCs, since in the absence of the positive regulator of p-SRC, and in the presence of negatively regulating NUMB [45], NOTCH1 expression is both decreased and simultaneously degraded.

C-myc, a downstream signaling target of NOTCH1, is also notably downregulated in sh*mda-9* PCSCs (Table 4). After DLL1-mediated activation of NOTCH1, the intracellular domain of NOTCH1, translocates into the nucleus and binds to the promoter region of the transcription factor RBPJK, which regulates *c-myc* expression [45]. Given the critical importance of C-myc in prostate cancer [56,57] stem cell self-renewal, maintenance, survival [58,59] and MDR1 expression [38], it is apparent that MDA-9-mediated C-myc expression are major contributors to the PCSC phenotype.

A prominent role of PCSCs in tumorigenesis is established [22,67] and we now show that control PCSCs injected subcutaneously into nude mice promote tumor formation (Figure 2B). However, kd of *mda-9* with Ad.5/3.sh*mda-9* infection in PCSCs result in substantially smaller tumors as compared to the sh*con* group (Figure 2B,C), indicating the importance of MDA-9 for PCSC-mediated tumorigenicity. 

**Figure 7:**
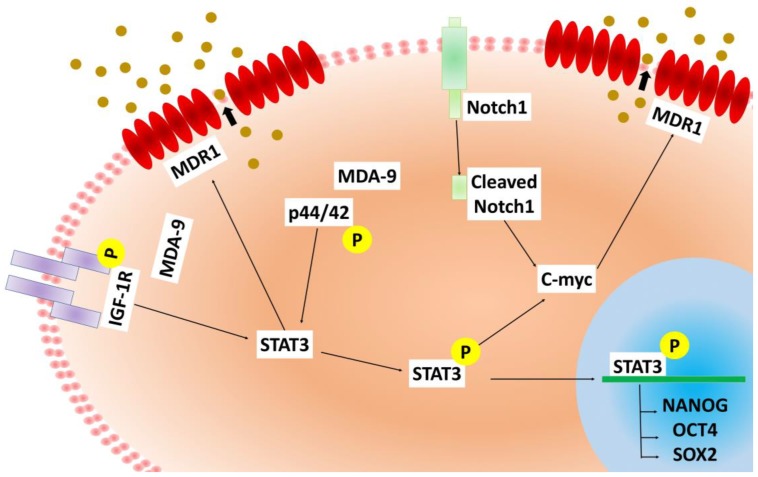
Schematic representation of MDA-9 mediated regulation of chemoresistance, in PCSCs. MDA-9 potentially regulates chemoresistance through IGF-1R/p44/42/STAT3/MDR1 and C-myc/MDR1 axis, and stem regulatory genes OCT4, NANOG and SOX-2 through STAT3.

The current therapeutic strategies for prostate cancer, such as androgen ablation, emphasize elimination of the majority of cells within the tumor. However, this often leads to therapy resistance in the majority of patients [4]. Thus, prostate cancer therapy can actually promote disease progression by potentially activating normally quiescent cancer stem cells to repopulate the tumor with androgen-independent cells [4]. Accordingly, it is important to develop therapeutics that can selectively target cancer stem cells, along with the more differentiated cancer cells. Detailed expression analysis of enriched populations of cancer stem cells provide improved identification of novel therapeutic targets [4,18]. Stem cells are dormant, long-lived, self-renewing cells that are protected both by location as well as resistance to multiple chemotherapeutic agents, which are usually anti-proliferative in nature. The microenvironment in the stem cell niche requires close examination as it may contribute significantly to the success or failure of a therapeutic treatment [4]. We now demonstrate that MDA-9 may be such a crucial element, which has a central regulatory role in the survival and maintenance as well as chemoresistance of PCSCs. MDA-9 physically interacts with IGF-1R, thereby regulating STAT3 phosphorylation at Tyr-705 [46,68]. By regulating STAT3 and C-myc, MDA-9 contributes to the observed resistance to docetaxel and trichostatin A (Figure 3 and Figure 4, Supplementary Figures S2, S3 and S5). MDR1 is one of the principal protectors of stem cells [40] and is also involved in inducing chemoresistance of cancer stem cells. This ATP-dependent efflux pump ABCB1 (MDR1), which encodes the membrane drug transporter P-glycoprotein, is a well described resistance mechanism for doxorubicin, paclitaxel and related taxane drugs [69,70,71]. An increased ABCB1 copy number results from a chromosomal amplification event at 7q11.2-21 and correlates with increased P-glycoprotein expression in paclitaxel-resistant cells from various cancers [72], with resulting drug-resistant phenotypes confirmed by ABCB1 overexpression and P-glycoprotein inhibitor studies [73]. We show that loss of MDA-9 significantly decreases MDR1 expression at both transcriptional as well as protein levels (Figure 5 and Figure 6), leading to increased caspase-mediated cell death post chemotherapeutic treatment (Figure 3). MDA-9 silencing through both genetic and pharmacological techniques promotes similar effects on MDR1 expression (Figure 5 and Figure 6). This loss of MDR1 in sh*mda-9* was regained by the expression of a constitutively active form of STAT3 (Figure 6B,C), indicating that MDA-9-mediated STAT3 signaling represents an important axis in PCSC chemoresistance. STAT3 signaling and *c-myc* are present in a feedback loop, suggesting that MDA-9 expression affects not only STAT-3 but also *c-myc*-mediated signaling. Inhibition of c-myc also decreased MDR1 expression (Supplementary Figure S5), adding another layer of regulation to MDA-9-mediated chemoresistance in PCSCs. MDA-9 regulates the expression of *c-myc* in PCSCs though the DLL1-NOTCH1-*c-myc* pathway [45] (Figure 7). This MDA-9-mediated pathway may possibly further regulate *c-myc*-mediated chemoresistance (Supplementary Figure S5). C-myc is a confirmed regulator of stem cells and is one of the key factors among the three Yamanaka factors essential for pluripotency of stem cells. Hence, MDA-9 regulates stemness and PCSC chemoresistance not only though STAT3 (Figure 4 and Figure 6B,C), but also through *c-myc*, connecting inherent resistance of cancer stem cells to chemotherapy with their capacity for self-renewal.

Our data establishes MDA-9 as a critical member of the complex, tightly regulated signaling network that regulates self-renewal, survival, progression and chemoresistance properties in PCSCs (Figure 7). MDA-9 may regulate stem-cell phenotypes in both normal prostate epithelial stem cells and PCSCs through similar pathways. However, PCSCs seem highly dependent on (“addicted to”) MDA-9 for proper functioning and survival, due to their highly elevated MDA-9 expression levels (Table 1), as compared to the normal prostate stem cells. Forced elevated expression of MDA-9 in normal prostate stem cells also increases their self-renewal and the overall stem cell population. MDA-9 controls the stem-phenotype on multiple molecular levels, emphasizing its pivotal role in PCSC functioning. The central transcriptional network of stem regulating genes, tumor-progressive capabilities, and the interconnected pathways crucial for PCSC survival, are all dependent on elevated levels of MDA-9. Since PCSC survival and maintenance are dependent on MDA-9, directly targeting MDA-9 expression or its interaction with downstream interacting partners through genetic or pharmacological approaches, may provide a unique opportunity to develop targeted therapies. This could utilize combinations of PDZ1i [42], a small molecule pharmacological inhibitor of MDA-9 protein–protein binding, with C-myc inhibitors to enhance sensitivity to standard chemotherapy. Since the use of MDR1 inhibitors has shown mixed clinical benefits and can result in severe adverse effects, this approach could be a more effective and less toxic way to effectively target PCSCs. This MDA-9-targeting approach may result in elimination of the cancer, as well as preventing recurrence. Although these studies were performed in androgen-independent prostate cancer cells, it would also be important to investigate MDA-9 inhibition in combination with hormonal therapeutics such as enzalutamide or abiraterone to overcome resistance to these drugs in PCSCs-derived from hormone-dependent as well as castrate-resistant tumors. The fact that MDA-9 targeting sensitizes androgen-independent PCSCs to therapy could have profound implications for neuroendocrine prostate cancer, a lethal and aggressive form of the disease, characterized by loss of androgen receptor (AR) signaling.

Cancer stem cells represent primary determinants of therapy resistance and recurrence. Recognizing the cells/tissues from which cancers initiate facilitates early detection and can possibly lead to the identification of targets to protect patients from morbidity and death. Conventional therapies are not adequate to eradicate cancer stem cells; therefore, predisposing to treatment failure and cancer recurrence [74]. Consequently, designing strategies which accomplish multiple endpoints provide a potential path toward enhanced cancer treatment. “Theranostic” cancer platforms represent one multifunctional approach, since they are designed to simultaneously facilitate both cancer diagnosis and treatment. Cancer stem cells enable resistance to therapy and cancer recurrence and must be eliminated to produce a fully effective cancer treatment. To achieve this objective, mechanisms of cancer stem cell maintenance and survival must be precisely defined [74]. Our studies show that PCSCs are critically dependent on (“addicted to”) MDA-9 for self-renewal and survival as well as chemoresistance highlighting the immense potential of using a “theranostic” approach focused on identifying PCSCs and simultaneously targeting MDA-9. If successful, this strategy could result in enhanced therapy of advanced prostate cancer and prevent cancer recurrence.

## 4. Materials and Methods

### 4.1. Cell Lines and Tissue Samples

Human prostate cancer cells DU-145 (ATCC, Manassas, VA, USA) and PC3-ML cells were cultured in DMEM supplemented with 10% fetal bovine serum and antibiotics. PC3-ML-Luc cells were obtained from Dr. Martin G. Pomper (Johns Hopkins Medical Institutions, Baltimore, MD, USA). ARCaP-M cells (Novicure Biotechnology, Birmingham, AL, USA) were cultured using MCAP medium supplemented with 10% fetal bovine serum and antibiotics (Novicure Biotechnology). Isolated non-stem prostate cancer cells (NSCC, Seattle, WA, USA), based on lack of expression of CD133 and CD44, were similarly cultured in monolayer culture. Normal immortal human prostate epithelial cells, RWPE-1, were obtained from ATCC and grown in Keratinocyte–SFM media supplemented with human recombinant Epidermal Growth Factor, Bovine Pituitary Extract (BPE) and antibiotics (Thermo Fisher Scientific, Waltham, MA, USA). The cumulative culture length of the cells was less than 6 months after being placed in culture. Early passage cells were used for all experiments. All the cell lines were routinely tested (every 6 months) for mycoplasma contamination using a mycoplasma detection kit from Sigma (St. Louis, MO, USA).

### 4.2. Isolation and Culture of Putative Human PCSCs and NSCCs

Human PCSCs and NSCCs were isolated from 3 different prostate cancer cell lines: DU-145, ARCaP-M, and PC3-ML. DU-145 (ATCC) and PC3-ML cells were cultured in monolayers using complete DMEM media. PC3-ML-Luc cells were obtained from Dr. Martin G. Pomper (Johns Hopkins Medical Institutions, Baltimore, MD, USA). ARCaP-M cells (Novicure Biotechnology) were cultured in monolayers using complete MCAP medium (Novicure Biotechnology). The cells were next cultured in ultra-low attachment T25 or T75 culture flasks (Corning, New York, NY, USA) in Essential 8 medium (Thermo Fisher Scientific) to form PCSC-enriched prostaspheres and studied prior to 5 passages. Prostaspheres were disassociated and then labeled with CD44 and CD133 antibody (Miltenyi Biotech, Auburn, CA, USA) as PCSC markers [75]. Stained cells were sorted through a BD Aria II sorting station. Cells highly expressing both CD44 and CD133 were selected as PCSCs while, cells lacking both were selected as non-stem cancer cells [9,20,75,76]. CD44^+^ CD133^+^ PCSC and CD44^−^ CD133^−^ NSCC populations were counted and collected for further culturing. Xenografted human PCSCs were isolated from mice and analyzed for cell surface markers by flow cytometry. All PCSCs were grown in ultra-low attachment plates and flasks with Essential 8 medium (Invitrogen, Carlsbad, CA, USA), and NSCCs were cultured in monolayer with complete DMEM medium, unless otherwise indicated.

### 4.3. Isolation and Culture of Primary Prostate Epithelial Stem Cells

Primary immortal normal human prostate epithelial cells (RWPE-1, ATCC) were cultured initially in T175 flasks (Corning) in Keratinocyte-SFM media (Thermo Fisher). The cells were grown in ultra-low attachment plates and flasks (Corning) using Essential 8 medium (Thermo Fisher Scientific) to enrich the stem cell population. The cells were stained with CD44, CD133 and α_2_β_1_ antibodies as prostate stem cell markers [75], sorted and further cultured under ultra-low attachment conditions.

### 4.4. Promoter Reporter Assays

2 × 10^5^ cells were infected with either Ad.5/3.sh*con* or Ad.5/3.sh*mda-9*. The cells were transfected post-infection with an RBPJK–responsive luciferase reporter construct using Lipofectamine 2000 [46]. Cells were lysed and the resultant relative luciferase activity was measured using a Dual-Luciferase Reporter Assay system (Promega, Madison, WI, USA) (manufacturer’s instructions). Luciferase activity was normalized to Renilla activity, and the data show the average of triplicates ± S.D.

### 4.5. Reverse Transcription Polymerase Chain Reaction

PCSCs were pelleted by centrifugation, washed twice with PBS and then total RNA was extracted utilizing TRIzol (Invitrogen) followed by purification of the resultant lysate using the RNeasy kit (Qiagen, Frederick, MD, USA). First-strand cDNA was synthesized with SuperScript III reverse transcriptase (Invitrogen). Quantitative PCR studies were performed using the TaqMan Gene expression assays (Invitrogen) using a ViiA 7 fast real-time PCR system (Applied Biosystems, Foster City, CA, USA). Relative gene expression was normalized to 18S expression (Invitrogen).

### 4.6. Western Blotting

Cells were washed with ice-cold PBS prior to lysis. All the samples were incubated on ice using lysis buffer (20 mM Tris-HCl (pH 7.5), 150 mM NaCl, 1 mM Na_2_EDTA, 1 mM EGTA, 1% Triton-100, 2.5 mM Sodium pyrophosphate, 1 mM β-glycerophosphate, 1 mM Na_3_VO_4_, 1 µg/mL Leupeptin). Protein samples were prepared after determining protein concentration, and then loaded onto 8% SDS-PAGE. Membranes were stained with primary antibodies, followed by HRP-conjugated secondary antibodies. Individual bands were detected using ECL (Thermo Scientific). The relative intensity values of all the proteins expressed in the samples were normalized against β-actin expression. For densitometry evaluation, X-ray films were scanned and evaluated with ImageJ software (National Institutes of Health [NIH], Bethesda, MD, USA). Antibody details are provided in the Supplemental Information.

### 4.7. Immunofluorescent Staining and Confocal Microscopy

Cells were fixed with 4% paraformaldehyde for 20 min at room temperature, permeabilized with 0.1% Triton X-100 for 5 min and blocked with 5% bovine serum for 1 h. NANOG, SOX2 and OCT4 staining was performed according to the manufacturer’s instructions (CST, Danvers, MA, USA). Nuclei were visualized with 1 µg/mL DAPI. Cells were imaged using laser confocal microscopy (Leica, Buffalo Grove, IL, USA). The images were analyzed by Zen software.

### 4.8. Live Cell Imaging 

Live cell images were obtained using a Zeiss (San Diego, CA, USA) Cell Observer SD spinning disk confocal microscope furnished with a Yokogawa CSU-X1A spinning disk, two Photometrics Evolve 512 cameras (16-bit), a high-resolution piezoelectric driven Z-stage, an acousto-optic tunable filter, four lasers (405 nm, multiline argon 458/488/514 nm, 561 nm, 635 nm), and a PeCon A stage incubation system. The stage and chamber conditions were optimized to remain constantly at 37 °C, with the CO_2_ level at 5%. The CSCs were stained with Cell tracker green CMFDA (Invitrogen). Sequential imaging was performed in green (488 nm excitation, 525/50 nm emission) channels. 

### 4.9. Live/Dead Cell Assay

Live/Dead cell imaging was performed to assess fluorescence based cell viability. Staining was performed as per the manufacturer’s directions (Invitrogen), which was followed by imaging utilizing laser confocal microscopy (Leica). The images were analyzed by Zen software.

### 4.10. Cell Proliferation

Cells were seeded at 1 × 10^5^ cells/mL in 6-well ultra-low attachment plates. Next, MTT reagent (7.5 mg/mL) in phosphate-buffered saline was added (10 μL/well), and the cultures were incubated at 37 °C for 30 min. The reaction was stopped by the addition of acidified Triton buffer (0.1 M HCl, 10% (*v*/*v*) Triton X-100; 50 μL/well), and the tetrazolium crystals were dissolved by mixing on a plate shaker at room temperature for 20 min as well as pipetting. The samples were measured at 595 nm and a reference wavelength of 650 nm. The results represent the mean ± S.E. of 5 wells from one experiment that is representative of experiments repeated at least three times. 

### 4.11. Flow Cytometry Sorting and Analysis

Cell surface markers CD44, CD133, DLL1, MDR1 and Annexin V were stained according to the manufacturer’s instructions, followed by flow cytometry analysis using BD DIVA.

### 4.12. Intracellular Flow Cytometry

Intracellular STAT3, p-STAT3, p44/42, p-p44/42, p-SRC, and Numb protein expression were analyzed by intra-cellular flow cytometry [61,62]. Cell fixation, permeabilization as well as primary and secondary antibody staining were executed according to the manufacturer’s instructions. Flow cytometry was performed and analyzed using BD DIVA.

### 4.13. Tumorigenicity Studies

All experiments and procedures using mice were approved by the Institutional Animal Care and Use Committee (IACUC) of Virginia Commonwealth University Richmond, VA, USA, protocol code: AM10183). For the subcutaneous xenograft model, athymic male NCr-nu/nu mice (National Cancer Institute–Bethesda, MD, USA) were used (*n* = 10 per group). 

1 × 10^5^ PCSCs were injected per mouse. Animals were closely monitored for tumor size, weight and volume, according to the VCU-IACUC approved protocol and the resultant data were evaluated. Once tumors in the control group reached 2000 mm^3^, the mice were euthanized and the tumors of all the groups were measured at the same time.

### 4.14. Peptide Blocking Studies

Control and treated PCSCs (1 × 10^5^ cells) were cultured in 6-well ultra-low attachment plates. NOTCH1 blocking peptide (Biovision, Exton, PA, USA) were used at a concentration of 10 μg/mL and incubated with cells for 48 h. After incubation, the cells were stained and analyzed for viability, spheroid size and structure. 

### 4.15. shRNA Knockdown

shRNA sequences were obtained from Qiagen using the following sequences: 5’-TTGACTCTTAAGATTATGTAA-3’ (sh*mda-9* #3) and 5’-TGGGATGGTCTTAGAATATTT-3’ (sh*mda-9* #4). Ad.5/3.sh*mda-9* was constructed as previously described [46] utilizing the following primer sequences: forward: 5’-GCCTGCTTTTATCTTTGAACATATTATTAAGCGAATGAAGCCTAGTATAATGAAAA GCCTAATGGACCACACCATTCCTGAG-3’ and reverse: 3’-CGGACGAAAATAGAAAC TTGTATAATAATTCGCTTACTTCGGATCATATTACTTTTCGGATTACCTGGTGTGGT AAGGACTC-5’.

### 4.16. Chemotherapeutic Studies

Docetaxel, STATTIC and Trichostatin-A (TSA) were obtained from Sigma. The PCSCs were serum-starved for 24 h and then treated with the drugs for 48 h at a 10 μM concentration (unless mentioned otherwise) prior to assessing the sensitivity of PCSCs to these drugs. DMSO treated cells were used as control. *In vitro* PDZ1i treatments occurred at 25 μM concentration. *In vivo* PDZ1i treatments occurred at 25 mg/kg body weight of the mice, 3 times a week for a month. 

### 4.17. Statistical Analysis

All experiments performed *in vitro, in vivo* and *ex vivo*, were analyzed statistically using the Student’s *t* test and ANOVA (Microsoft Excel, 15.37 Redmond, WA, USA). Pearson’s correlation coefficient (R) and coefficient of determination (R^2^) were calculated for correlation analysis. All statistical tests were two-sided, and *p* values ≤ 0.05 and ≤ 0.01 were considered to be significant and highly significant, respectively.

## Figures and Tables

**Table 1:** Expression of *mda-9* and stemness genes in non-stem prostate cancer cells and prostate cancer stem cells.

Cell Line	DU-145		ARCaP-M		PC3-ML	
Genes	Non-Stem Cancer Cell	Cancer Stem Cell	Non-Stem Cancer Cell	Cancer Stem Cell	Non-Stem Cancer Cell	Cancer Stem Cell
*mda-9*	1 ± 0.05	3.48 ± 0.10	1 ± 0.20	8.30 ± 0.07	1 ± 0.07	5.67 ± 0.25
Stemness genes						
*Nanog*	1 ± 0.03	12.50 ± 0.02	1 ± 0.02	8.64 ± 0.11	1 ± 0.14	13.88 ± 2.0
*Sox2*	1 ± 0.20	2.94 ± 0.04	1 ± 0.32	2.49 ± 0.06	1 ± 0.19	4.48 ± 1.93
*Oct4*	1 ± 0.07	20.00 ± 0.01	1 ± 0.11	5.51 ± 0.01	1 ± 0.22	13.01 ± 0.45
*c-myc*	1 ± 0.15	2.44 ± 0.06	1 ± 0.09	2.39 ± 0.05	1 ± 0.03	3.93 ± 1.7

**Table 2:** Effect of *mda-9* expression on CSC populations in prostate cancer cells.

Cell Line	CSC Population (%) sh*con*	CSC Population (%) sh*mda-9*
DU-145	40.3 ± 5.2	11 ± 5.5
PC3-ML	15.4 ± 3.5	3.5 ± 1.2
ARCaP-M	29.8 ± 3.7	9.9 ± 2.4

**Table 3:** Effect of knockdown of *mda-9* (sh*mda-9*) on the expression of stemness genes in prostate cancer cell lines.

Genes	DU-145		ARCaP-M		PC3-ML	
	Sh*con*	Sh*mda-9*	Sh*con*	Sh*mda-9*	Sh*con*	Sh*mda-9*
*mda-9*	1 ± 0.07	0.2 ± 0.15	1 ± 0.05	0.3 ± 0.07	1 ± 0.06	0.1 ± 0.12
Stemness genes						
*Nanog*	1 ± 0.02	0.55 ± 0.04	1 ± 0.03	0.10 ± 0.04	1 ± 0.02	0.15 ± 0.02
*Sox2*	1 ± 0.06	0.32 ± 0.00	1 ± 0.01	0.26 ± 0.03	1 ± 0.08	0.10 ± 0.05
*Oct4*	1 ± 0.11	0.14 ± 0.04	1 ± 0.25	0.10 ± 0.01	1 ± 0.05	0.03 ± 0.00
*c-myc*	1 ± 0.01	0.17 ± 0.03	1 ± 0.02	0.05 ± 0.01	1 ± 0.02	0.09 ± 0.00

**Table 4:** Effect of *mda-9* knockdown (sh*mda-9*) on gene expression in prostate cancer cell lines.

% Expression	DU-145 sh*con*	DU-145 sh*mda-9*	ARCaP-M sh*con*	ARCaP-M sh*mda-9*	PC3-ML sh*con*	PC3-ML sh*mda-9*
Protein						
P-STAT3	62.3 ± 9.8	15.4 ± 4.1	6.5 ± 0.6	2.2 ± 0.3	41.3 ± 7.6	29.6 ± 3.1
STAT3	85.1 ± 8.6	87.4 ± 2.7	24.6 ± 1.9	25.9 ± 5.2	62.7 ± 7.9	65.7 ± 4.6
P-SRC	68.2 ± 6.5	11.5 ± 5.3	37.9 ± 5.6	12.6 ± 3.8	53.9 ± 4.2	37.1 ± 2.7
SRC	77.4 ± 5.1	75.2 ± 4.5	39.4 ± 5.8	34.1 ± 3.5	69.9 ± 6.7	63.3 ± 5.1
P-p44/42	18.8 ± 4.4	1.4 ± 0.07	21.2 ± 4.7	6.4 ± 0.6	6.4 ± 1.5	1.7 ± 0.2
p44/42	21.4 ± 2.9	4.8 ± 2.9	21.6 ± 9.5	11.9 ± 2.9	16.2 ± 2.4	6.5 ± 1.2
P-IGF1R	20.2 ± 5.3	9.7 ± 2.5	18.5 ± 2.9	10.5 ± 3.1	7.2 ± 1.1	1.8 ± 0.4
IGF1R	35.7 ± 4.9	36.9 ± 7.2	34.8 ± 7.4	36.2 ± 4.4	25.6 ± 5.7	27.4 ± 3.2
NOTCH1	94.3 ± 4.7	31.5 ± 8.5	49.7 ± 9.1	36.2 ± 2.6	74.7 ± 8.8	36.4 ± 4.6
DLL1	41.8 ± 8.2	13.7 ± 2.6	44.5 ± 12.6	32.9 ± 3.3	49.4 ± 6.9	27.6 ± 2.6
Numb	22.3 ± 4.8	44.3 ± 5.9	0.5 ± 0.1	7.3 ± 1.7	9.81 ± 2.4	19.5 ± 3.8
C-Myc	53.8 ± 2.2	25.4 ± 3.5	60.6 ± 9.6	39.6 ± 7.6	53.0 ± 3.1	37.8 ± 1.5

## Supplementary Materials

The following are available online at https://www.mdpi.com/2072-6694/12/1/53/s1, Figure S1. Effect of *mda-9* overexpression on PCSCs. Figure S2: Effect of kd of *mda-9* on PCSCs. Figure S3. MDA-9-mediated STAT3 regulation, and its downstream effects in PCSCs. Figure S4. (A) Image J analysis of the Western blots shown in Figure 5C to quantify MDR1 expression. (B) Expression of *mda-9* in shcontrol, *mda-9* kd and *mda-9* kd PCSCs overexpressing MDR1. Figure S5: MDA-9 regulates chemoresistance in PCSCs though the cmyc/MDR1 axis. Figure S6: *mda-9* regulates PCSC survival through the NOTCH1 pathway. Peptide blocking studies to elucidate the effect of Notch1 blocking peptide (NBP) on PCSC viability; live cells are green, dead cells are red.
