# Supplementary material for: MDA-9/Syntenin (SDCBP) Is a Critical Regulator of Chemoresistance, Survival and Stemness in Prostate Cancer Stem Cells
Source: Cancers (Basel). 2019 Dec 23;12(1):53. doi: 10.3390/cancers12010053 (PMC7017101; doi:10.3390/cancers12010053)
Supplement: Supplementary file 1 [file cancers-12-00053-s001.zip › cancers-665121-supplementary/cancers-665121-suppl-final/Supplementary figures.pdf]

cancers

Article

MDA-9/Syntenin (SDCBP) Is a Critical Regulator of Chemoresistance, Survival and Stemness in Prostate Cancer Stem Cells

Sarmistha Talukdar [1,2], Swadesh K. Das [1,2,3], Anjan K. Pradhan [1,2], Luni Emdad [1,2,3], Jolene J. Windle [1,2,3], Devanand Sarkar [1,2,3] and Paul B. Fisher [1,2,3,*]

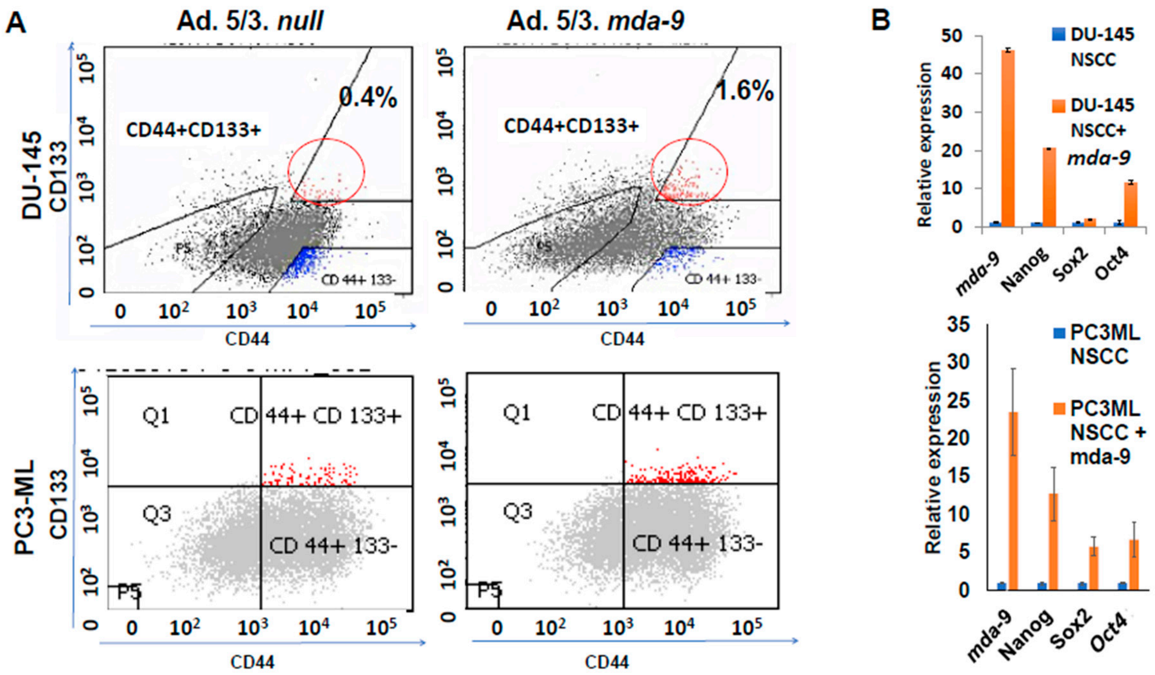

Figure S1. Effect of *mda-9* overexpression on PCSCs. Effect of overexpression of *mda-9* in prostate cancer stem cells and non-stem cancer cells on (A) stem populations as assayed by cell surface stemness markers and (B) stem regulatory molecule (NANOG, OCT4 and SOX2) expression.

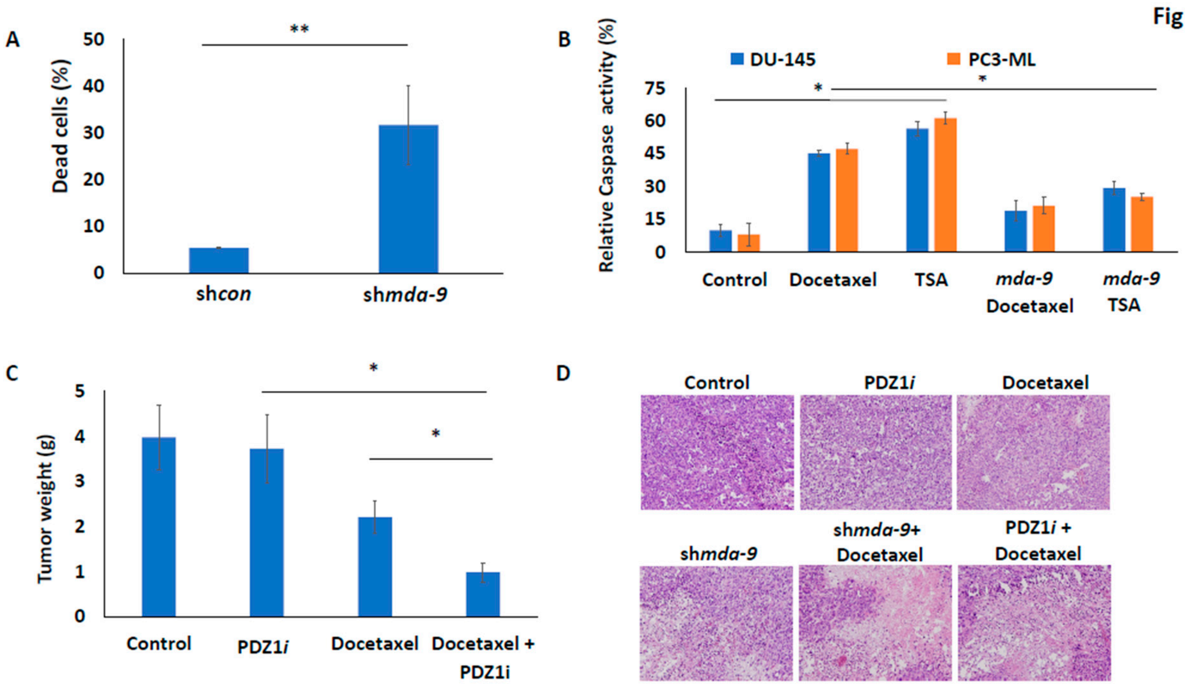

Figure S2. Effect of kd of *mda-9* on PCSCs. KD of *mda-9* enhances PCSC cell death (A). PCSC sensitivity to docetaxel and TSA treatment *in vitro* and effect of *mda-9* overexpression on PCSC sensitivity to chemotherapeutics. (B) Effect of PDZ1i (pharmacological inhibitor of MDA-9) on docetaxel sensitivity of tumors measured by tumor weight (C) and analyzed by histopathology (D). Bars represent SEM. *P<0.05, using student t-test and ANOVA.

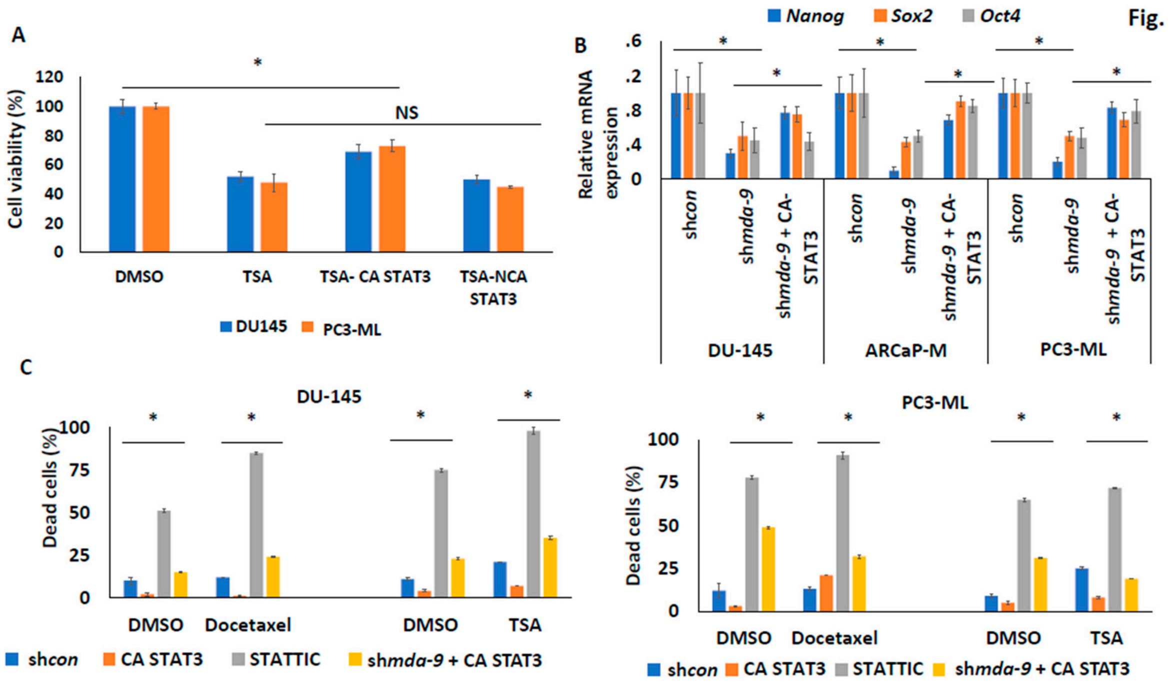

Figure S3. MDA-9-mediated STAT3 regulation, and its downstream effects in PCSCs. (A) STAT3 signaling through MDA-9 regulates chemosensitivity to the HDAC inhibitor TSA. (B) STAT3 activation by MDA-9 regulates stem regulatory molecules including OCT4, SOX2 and NANOG. (C) Image analysis data showing the role of STAT3 in MDA-9-based chemoresistance to Docetaxel and TSA in DU-145 and PC3-ML PCSCs. Bars represent SEM. *P<0.05, using student t-test and ANOVA. NS depicts results that are not statistically significant.

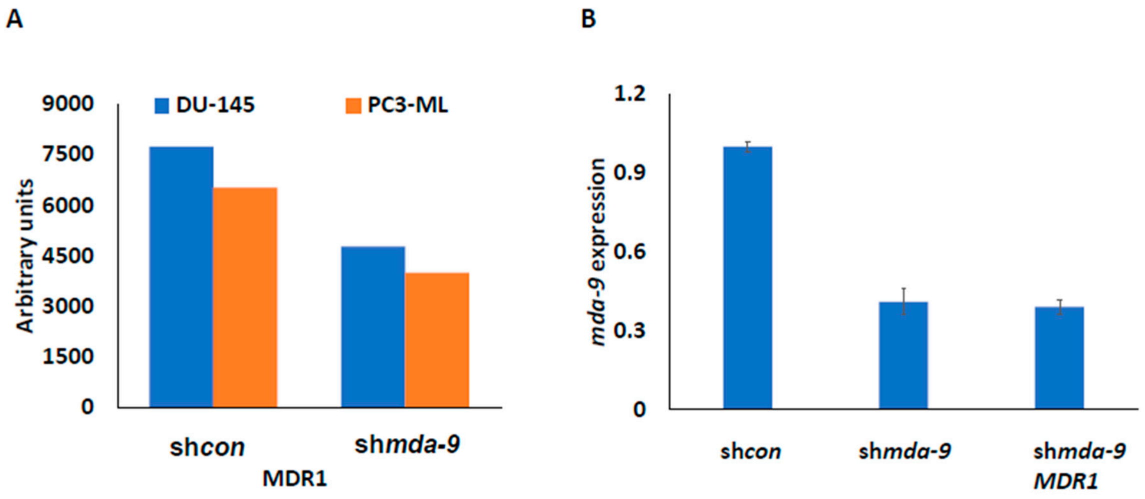

Figure S4. (A) Image J analysis of the Western blots shown in Figure **5C** to quantify MDR1 expression. (B) Expression of *mda-9* in shcontrol, *mda-9* kd and *mda-9* kd PCSCs overexpressing *MDR1*.

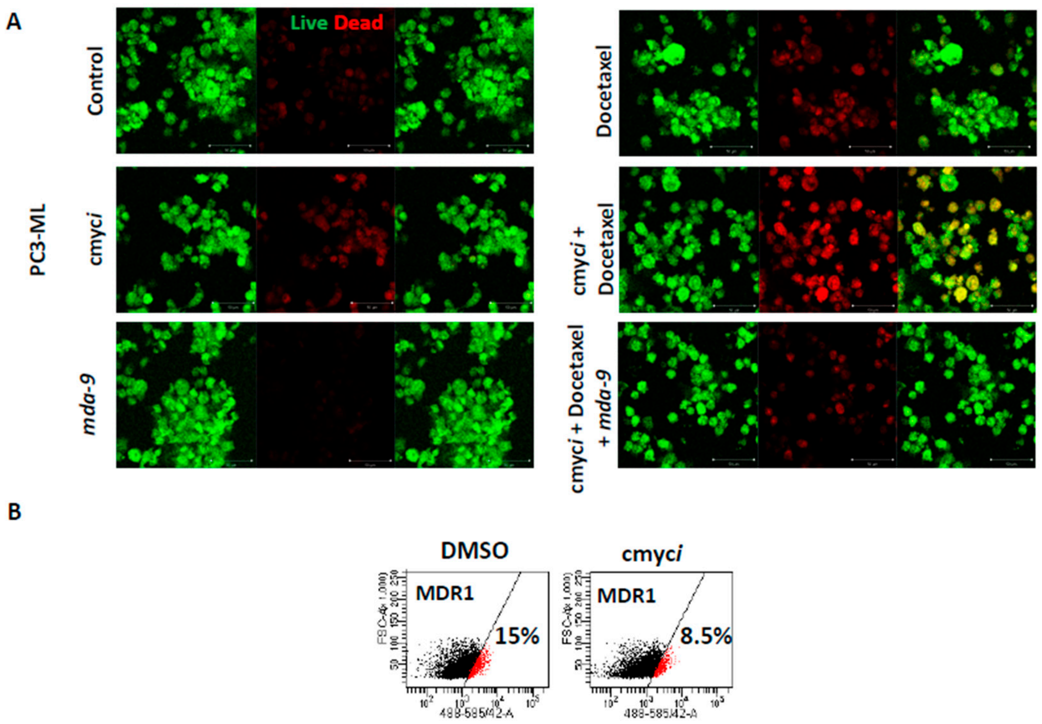

Figure S5. MDA-9 regulates chemoresistance in PCSCs though the cmyc/MDR1 axis. (A) Live/Dead analysis of PCSC sensitivity to Docetaxel after cmyc*i*, Docetaxel, *mda-9* overexpression treatment alone and in combination. (B) Effect of cmyc inhibition on MDR1 expression.

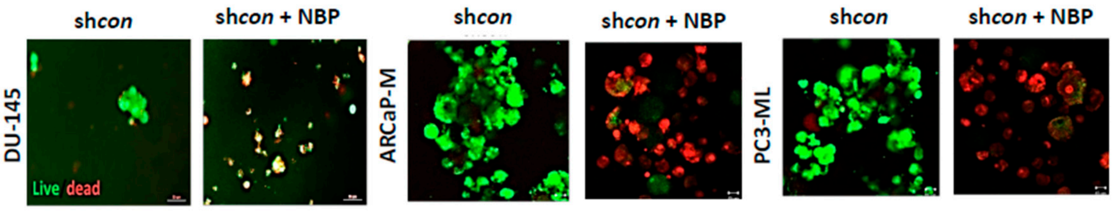

Figure S6. *mda-9* regulates PCSC survival through the NOTCH1 pathway. Peptide blocking studies to elucidate the effect of Notch1 blocking peptide (NBP) on PCSC viability; live cells are green, dead cells are red.
