# Supplementary material for: MDA-9/Syntenin (SDCBP) Is a Critical Regulator of Chemoresistance, Survival and Stemness in Prostate Cancer Stem Cells
Source: Cancers (Basel). 2019 Dec 23;12(1):53. doi: 10.3390/cancers12010053 (PMC7017101; doi:10.3390/cancers12010053)
Supplement: Supplementary file 1 [file cancers-12-00053-s001.zip › cancers-665121-supplementary/cancers-665121-suppl-final/Supplementary materials.pdf]

cancers

MDPI

Supplementary Materials and Methods

Plasmids

Small hairpin (sh)RNA for mda-9/syntenin (sh*mda-9*/syntenin) was created with pSilencer hygro expression vectors according to the manufacturer's protocol (Ambion). The specific hairpin small interfering (si)RNA oligonucleotides sequences are:

sense

5′GATCCGCGGATGGCACCAAGCATTTTCAAGAGAAATGCTTGGTGCCATCCGCTTTT

TTGGAAA-3′

and antisense

5′AGCTTTTCCAAAAAAGCGGATGGCACCAAGCATTTCTCTTGAAAATGCTTGGTGCC

ATCCGCG-3′

The oligo nucleotides were annealed and ligated to pSilencer vector by T4 DNA ligase. Alternate siRNA sequences were obtained through Qiagen with the following sequences: 5′-TTGACTCTTAAGATTATGTAA-3′ (si*mda-9* #3). sh*mda-9* resistant *mda-9/syntenin* plasmid was created using the following primer sequences:

forward

5′-GCCTGCTTTTATCTTTGAACATATTATTAAGCGAATGAAGCCTAGTATAATGAAAA

GCCTAATGGACCACACCATTCCTGAG-3′

and reverse:

3′-CGGACGAAAATAGAAACTTGTATAATAATTCGCTTACTTCGGATCATATTACTTTT

CGGATTACCTGGTGTGGTAAGGACTC-5′.

These plasmids were cleaved and transfected into HEK-293 cells to obtain the corresponding Ad.5/3-based vectors. The viruses were then expanded using HEK-293 cells and purified by cesium chloride double ultracentrifugation (Beckman SW28 rotor) using standard protocols (OD260 Inc., Boise, ID). The infectious viral particles were titered by plaque assay as described [46].

RT-PCR Thermofisher Taqman Probes

mda-9 Hs01045460_g1
myc Hs00153408_m1
Nanog Hs04399610_g1
Sox2 Hs00415716_m1
Oct4 Hs04260367_gH
CD133 Hs01009250_m1
Notch1 Hs01062014_m1
18S Hs99999901_s1
ABCB1 Hs00184500_m1
ABCC1 Hs01561483_m1
ABCC2 Hs00960488_m1

ABCC3 Hs00358656_m1
ABCC5 Hs00981089_m1
ABCG2 Hs01053790_m1
The interrogated sequence for each probe can be obtained at the Thermofisher Gene expression assays (Taqman) site.

PCR conditions

SuperScript™ Double-Stranded cDNA Synthesis Kit from Invitrogen was used to generate cDNA. We used the protocol as suggested by the company. 1µg of Total RNA was used per 20µl cDNA reaction. The tube was placed in the PCR machine programmed as follows: 25°C - 10 min, 50°C - 50 min, 85°C - 5 min. For RT-PCR, the cDNA reaction was diluted 1:10 with DEPC water and use 5µl as template for a 20µl RT-PCR reaction. RT-PCR was performed using ViiA 7 fast real-time PCR system, using the company protocol. These PCR conditions included polymerase activation at 95°C for 4 min followed by 30 cycles at 95°C for 30 sec, 63°C for 30 sec, and 72°C for 30 sec.

Virus Infections

Viral infections were performed as described previously [46].

Transfections

The cells were transfected with an constitutively active CA-STAT3 construct with Lipofectamine 2000 as described [45].

Immunohistochemistry

The mice were monitored and euthanized according to an approved protocol, and the tumors were dissected. The tumor samples from all the groups of animals were fixed in phosphate-buffered formalin and paraffin sections were prepared using standard histology protocols. Paraffin-embedded sections were dewaxed and rehydrated through incubations in xylene and a gradient series of alcohol. Antigen retrieval was performed in 10 mM citric acid (pH 6.0) with microwave treatment for 20 min. Endogenous hydrogen peroxidase was quenched by 3% (vol/vol) H_2O_2 treatment for 20 min. Nonspecific binding sites were blocked with a solution of 5% (vol/vol) normal sera, and the sections were incubated with antibody overnight. The sections were incubated with biotinylated secondary antibodies and subsequently with avidin–biotin complex peroxidase (Vector Elite; Vector Laboratories). Colorimetric reactions were developed by DAB substrate (0.02% DAB (3,3′-diaminobenzidine), 0.005% hydrogen peroxide) treatment followed by 10% (vol/vol) Harris hematoxylin counterstaining. Hematoxylin & eosin staining was also conducted following a standard protocol as described earlier [46]. The images were analyzed under an Olympus BX41 microscope system equipped with DP25 digital camera and software.

Antibodies

CD44, CD133 were obtained from Miltenyi Biotech. MDA-9 antibody was obtained from Alpha-Diagnostic International (San Antonio, TX). IGFR, P-IGFR, P-STAT3, STAT3, Beta-actin, and MDR1 antibodies were obtained from Cell Signaling (Beverly, MA).
