# Supplementary figures and images for: MDA-9/Syntenin (SDCBP) Is a Critical Regulator of Chemoresistance, Survival and Stemness in Prostate Cancer Stem Cells
Source: Cancers (Basel). 2019 Dec 23;12(1):53. doi: 10.3390/cancers12010053 (PMC7017101; doi:10.3390/cancers12010053)

Figure 5C

IB : anti-MDR1

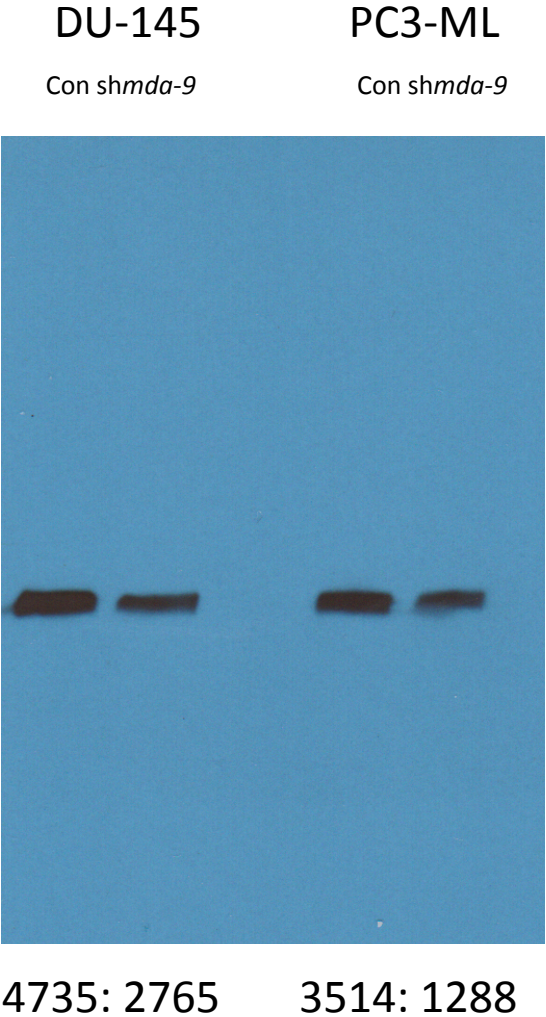

IB : anti-MDA-9

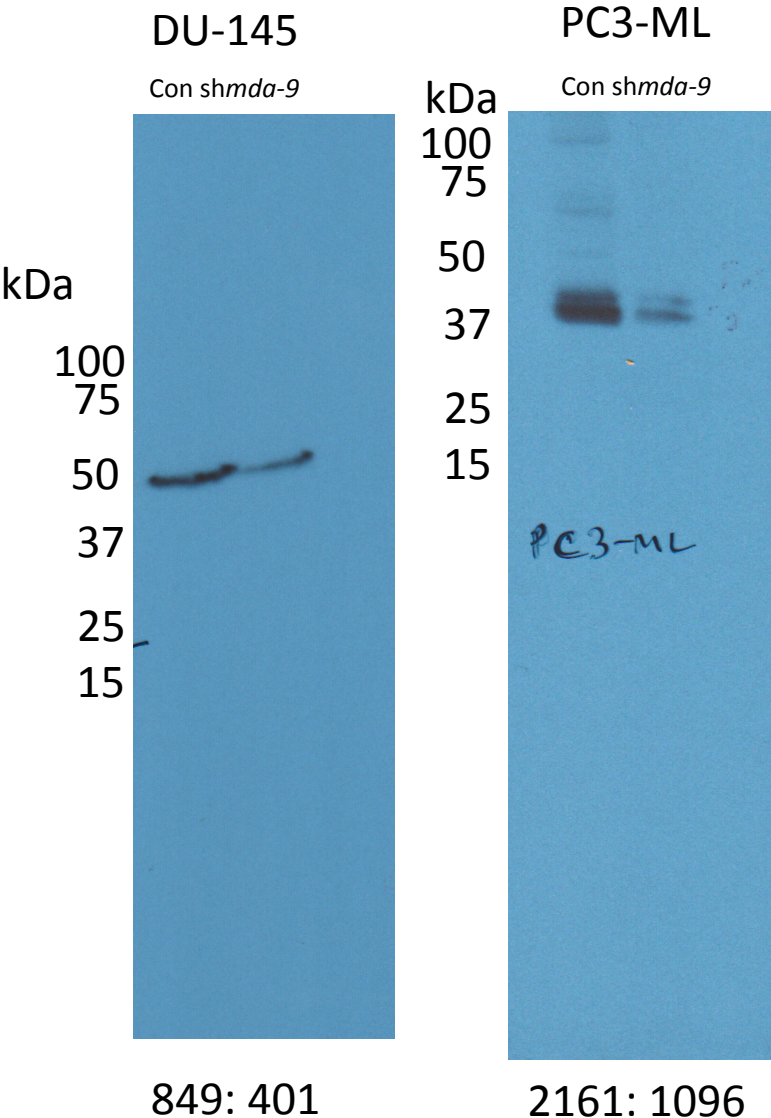

IB : anti-Beta actin

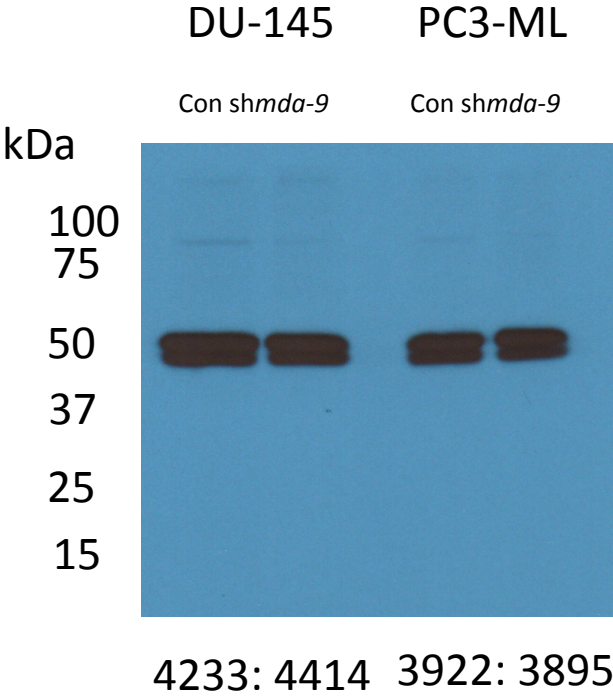

Figure 6B

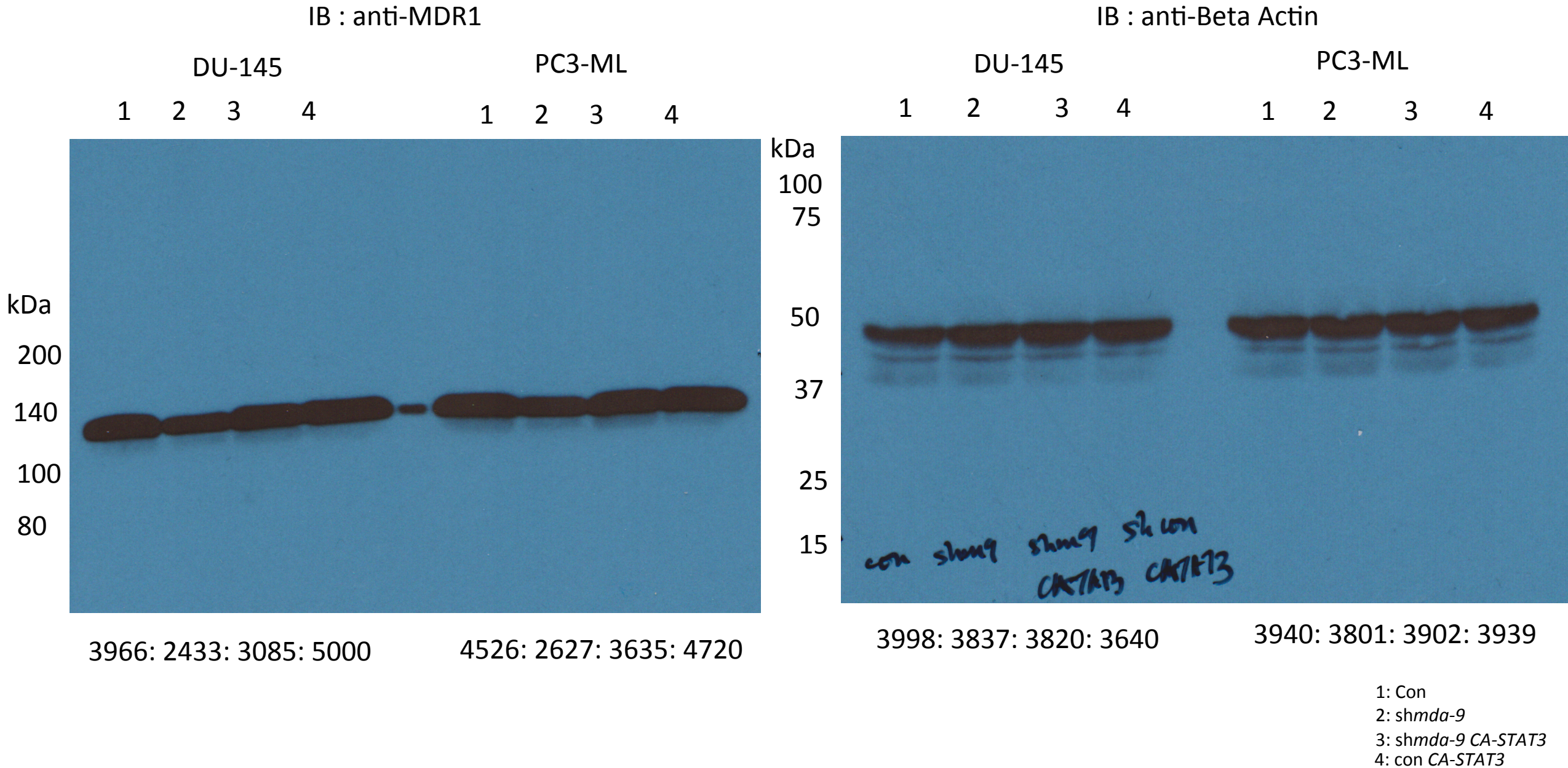

Supplement: Supplementary file 1 [file cancers-12-00053-s001.zip › cancers-665121-supplementary/cancers-665121-suppl-final/Western blots figures.pdf]
